# Dataset of human intracranial recordings during famous landmark identification

**DOI:** 10.1038/s41597-022-01125-8

**Published:** 2022-01-31

**Authors:** Oscar Woolnough, Cihan M. Kadipasaoglu, Christopher R. Conner, Kiefer J. Forseth, Patrick S. Rollo, Matthew J. Rollo, Vatche G. Baboyan, Nitin Tandon

**Affiliations:** 1 Vivian L. Smith Department of Neurosurgery, McGovern Medical School at UT Health Houston, Houston, TX 77030 United States of America; 2 Texas Institute for Restorative Neurotechnologies, University of Texas Health Science Center at Houston, Houston, TX 77030 United States of America; 3 Memorial Hermann Hospital, Texas Medical Center, Houston, TX 77030 United States of America

**Keywords:** Visual system, Long-term memory

## Abstract

For most people, recalling information about familiar items in a visual scene is an effortless task, but it is one that depends on coordinated interactions of multiple, distributed neural components. We leveraged the high spatiotemporal resolution of direct intracranial recordings to better delineate the network dynamics underpinning visual scene recognition. We present a dataset of recordings from a large cohort of humans while they identified images of famous landmarks (50 individuals, 52 recording sessions, 6,775 electrodes, 6,541 trials). This dataset contains local field potential recordings derived from subdural and penetrating electrodes covering broad areas of cortex across both hemispheres. We provide this pre-processed data with behavioural metrics (correct/incorrect, response times) and electrode localisation in a population-normalised cortical surface space. This rich dataset will allow further investigation into the spatiotemporal progression of multiple neural processes underlying visual processing, scene recognition and cued memory recall.

## Abstract (summary)

**Table:** 

Measurement(s)	electrophysiology data
Technology Type(s)	Electrocorticography
Factor Type(s)	visual scenes
Sample Characteristic - Organism	Homo sapiens

Machine-accessible metadata file describing the reported data: 10.6084/m9.figshare.16727317

## Background & Summary

Analysing and identifying previously encountered scenes and landmarks requires an interplay of activity between visual and memory regions of the brain. Category selective scene processing pathways exist across higher visual cortex^1–3^ and specialised, distributed memory networks exist for recall of scene and location specific information^4–8^. Neurobiological studies of higher-level image and scene processing in humans are usually derived using fMRI which, given its relatively low temporal resolution, provides a representation of the locations of neural processes but not their dynamics. Visual recognition and identification is accomplished by a combination of feedforward and feedback interactions between multiple substrates. While the traditional view of visual processing follows a hierarchical feedforward structure from low level visual features to high level category representations^9^, recurrent inputs from high level regions modulate activity in early visual regions enhancing sensitivity to certain higher order features^10–12^. Therefore, information about the temporal progression of feature sensitivity is required to disentangle these properties within visual processing networks and refine existing models of scene processing.

Given the special circumstances that it takes to obtain them, intracranial recordings remain a relatively rare but unique window into local and interactional cortical dynamics. We used a famous landmark identification task to probe multiple aspects of scene processing pathways; including visual processing of the scene stimuli, memory recall of the specific item and articulation of the response. These data were collected in a large population of patients (52 datasets) with a large number of electrodes implanted across both hemispheres (n = 6,775) allowing broad coverage across multiple cortical regions. Some of these data have previously been used to elaborate category selective cortical organisation in the ventral occipitotemporal cortex^13–15^, and memory recall in medial parietal cortex and medial temporal lobe^15^. Given the very rich nature of this dataset there remains multiple aspects of vision processing and memory recall that remain unaddressed and can be probed using these data.

## Methods

### Participants

50 patients (24 male, 18–51 years, 11 left-handed) participated in the experiments after giving written informed consent. Participants consented for anonymised data to be distributed for research use. All participants were semi-chronically implanted with intracranial electrodes for seizure localisation of pharmaco-resistant epilepsy. Two patients participated in the experiments twice (TA632/TA632C, TS060B/TS060C), during two separate electrode implantations (52 implantations total), with a separation of approximately two years between implants. Participants with significant additional neurological history (e.g. previous resections, MR imaging abnormalities such as malformations or hypoplasia) were excluded. All experimental procedures were reviewed and approved by the Committee for the Protection of Human Subjects (CPHS) of the University of Texas Health Science Center at Houston as Protocol Number HSC-MS-06-0385.

### Electrode implantation and data recording

Data were acquired from either subdural grid electrodes (SDEs; 10 patients) or stereotactically placed depth electrodes (sEEGs; 42 patients)^15^. SDEs were subdural platinum-iridium electrodes embedded in a silicone elastomer sheet (PMT Corporation; top-hat design; 3 mm diameter cortical contact), and were surgically implanted via a craniotomy^16–18^. sEEGs were implanted using a Robotic Surgical Assistant (ROSA; Medtech, Montpellier, France)^19,20^. Each sEEG probe (PMT corporation, Chanhassen, Minnesota) was 0.8 mm in diameter and had 8–16 electrode contacts. Each contact was a platinum-iridium cylinder, 2.0 mm in length and separated from the adjacent contact by 1.5–2.43 mm. Each patient had 12–20 such probes implanted. Following surgical implantation, electrodes were localised by co-registration of pre-operative anatomical 3 T MRI and post-operative CT scans in AFNI^21^. Electrode positions were projected onto a cortical surface model generated in FreeSurfer^22^, and displayed on the cortical surface model for visualisation^17^. Population-level cortical maps were generated using a surface-based co-registration to address sparse-sampling, as well as intra- and inter-subject data variability, including the topological variability arising from each subject’s own complex cortical geometry. In brief, individual cortical surface models were inflated and warped to match the folding patterns on a spherical template mesh, derived from a population atlas (Colin 27), and then resampled to a new standardised mesh providing one-to-one vertex correspondence between vertex indices and anatomical locations across subjects^23,24^.

Intracranial data were collected during research experiments starting on the first day after electrode implantation for sEEGs and two days after implantation for SDEs. Data were digitised at 2 kHz using the NeuroPort recording system (Blackrock Microsystems, Salt Lake City, Utah; acquisition filter 0.3–500 Hz), imported into Matlab, initially referenced to the white matter channel used as a reference for the clinical acquisition system and visually inspected for line noise, artifacts, and epileptic activity. Electrodes with excessive line noise or localised to sites of seizure onset were excluded. Trials contaminated by inter-ictal epileptic spikes are marked.

### Stimuli and experimental design

**Fig. 1:**
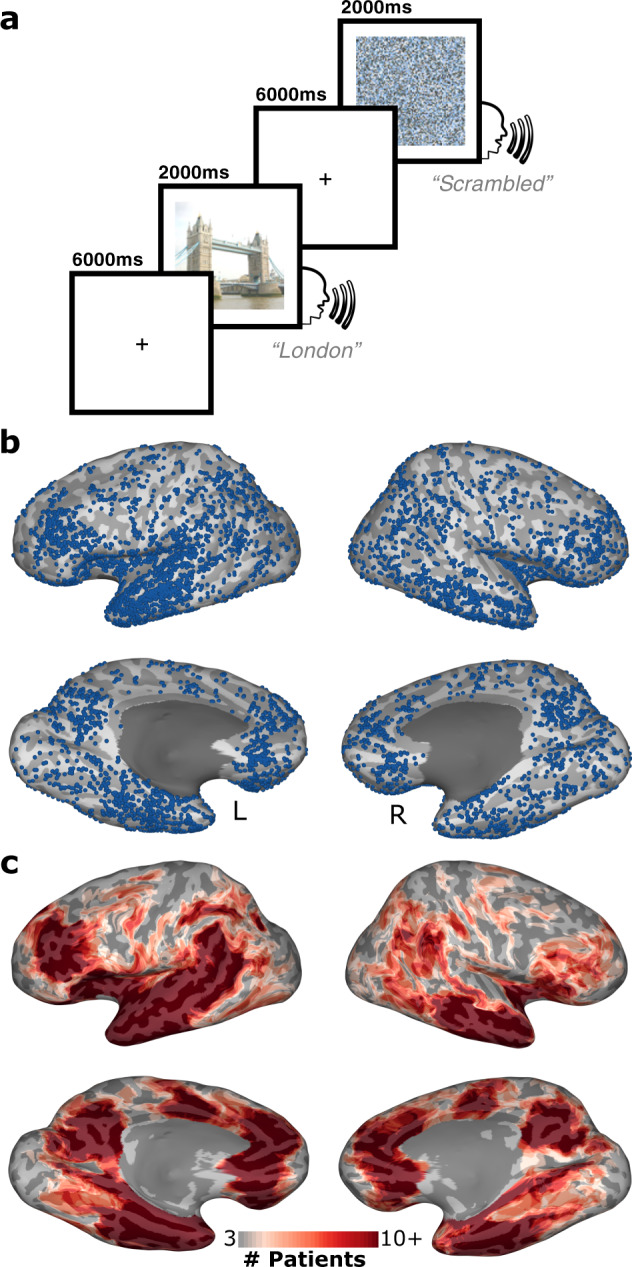
Experimental Design and Electrode Coverage. (**a**) Schematic representation of the landmark identification task. Participants were shown either coherent images of famous landmarks or spatially scrambled versions of the images. (**b**) Individual electrode locations (6,775 electrodes) and (**c**) representative coverage map (52 implantations).

Stimuli were presented using Python v2.7 at a size of 500 × 500 pixels on a 2,880 × 1,800, 15.4” LCD screen positioned at eye-level, 2–3′ from the patient (~7.5° visual angle). Each stimulus was displayed for 2,000 ms with an inter-stimulus interval of 6,000 ms. Participants were presented with colour photos of famous landmarks (scenes) and asked to verbally recall their location (Fig. 1a)^13–15^. Stimuli were presented in one recording session, containing presentation of 140–160 images, consisting of a mix of coherent images and their spatially scrambled versions in a pseudorandom order. In trials with scrambled scenes the patients were asked to respond with “scrambled”.

### Audio recordings

Continuous audio recordings were carried out during all experiments with an omnidirectional microphone (30–20,000 Hz response, 73 dB SNR, Audio Technica U841A) placed within 2 feet of the patient, and adjacent to the presentation laptop. These recordings were analysed offline to manually isolate articulatory onsets and assess patient responses. As voice recordings are biometric data protected under HIPAA, this dataset is released only with the manually picked articulation onset times and a binary correct/incorrect variable to denote the accuracy of the answer given. Incorrect responses included either an absence of speech, stating they did not recognise the object or incorrectly identifying the object.

### Signal analysis

Example analyses presented here were performed by first bandpass filtering raw data of each electrode into broadband gamma activity (BGA; 70–150 Hz). A frequency domain bandpass Hilbert transform (paired sigmoid flanks with half-width 1.5 Hz) was applied and the analytic amplitude was smoothed (Savitzky - Golay finite impulse response, 3rd order, frame length 301 ms). BGA is presented here as percentage change from baseline level, defined as the period −500 to −100 ms before each stimulus presentation.

## Data Records

The dataset^25^ was released in the Neurodata Without Borders (NWB)^26,27^ Version 2.2.5 format and is available online from the Data Archive for the BRAIN Initiative (DABI). The dataset is organised according to iEEG-BIDS^28^. Data from each recording session, including their associated pre-processing metadata and electrode localisations were saved in a single HDF5 file (.nwb). Standard NWB file structure was used which comprises a hierarchical structure^29^. Top level data groups include subject information, electrode information, time series data and trial information (Table 1).

**Table 1:** Summary of the data structure.

**Surface Information (general_subject.corticalsurfaces)**	
vertices	3D coordinates of the vertices of the cortical surface
faces	Vertex triplets that form a face of the cortical surface
HCP	HCP atlas parcel index and area name^30^
curv	Local curvature of the standard pial surface. >0 – Sulcus, <0 – Gyrus
**Electrode Information (general_extracellular_ephys_electrodes)**	
x,y,z	3D electrode location in standard inflated surface space
vertex	Assigned vertex in standard surface space
hemi	Brain hemisphere assigned to
HCP	HCP atlas parcel index^30^
location	HCP atlas area name^30^
good	1 – Usable electrode, 0 – Removed electrode, values set to NaN
zone	Electrode zone localisation. White, Gray, CSF, SDE.
pial_dist	Euclidean distance from the pial surface in mm
**Trial Information (intervals_trials)**	
start_time	Event markers
articulation	0 – Stimulus onset locked, 1 – Articulation onset locked
rxn_time	Time from stimulus onset to articulation onset (seconds)
good	1 – Usable trial, 0 – Excluded trial due to interictal spikes or task disruption
correct	1 – Correctly answered trial, 0 – Incorrectly answered trial
scrambled	0 – Coherent image presented, 1 – Scrambled image presented
stimulus	File name of presented visual stimulus

### Subject information (general_subject)

This data group contains basic patient demographics including age, sex and implant type.

### Cortical surfaces (general_subject.corticalsurfaces)

In order to allow cross-individual consolidation of data, all electrodes have been mapped onto a standardised cortical surface. Each electrode is assigned to a vertex of the standard population surface. We have provided both inflated (std_inflated) and pial (std_pial) versions of this standard surface. By knowing the hemisphere and vertex assignment of an electrode then the 3D location can be determined for either representation. The cortical surface map contains the vertices and faces of each hemisphere. For each vertex we provide a Human Connectome Project parcellation map assignment^30^ (HCP) and the local curvature value of the pial surface (curv).

### Electrode information (general_extracellular_ephys_electrodes)

For each electrode we provide a 3D location in standard inflated space (x,y,z). The assigned vertex of each electrode (vertex) and its hemispheric assignment (hemi) are also included. Based on this vertex assignment each electrode is given an HCP label (HCP, location). Electrodes with excessive line noise or localised to sites of seizure onset were marked (good = 0) and the time series data excluded.

### Time series data

Each participant has continuous time series data, recorded from each of the electrodes for the duration of the experimental session. Time series data is provided referenced to the original clinical white matter reference electrode (either electrode 5 or 6) and grounded to electrode 7. We have included a virtual channel representing the common average of the good channels (labelled CAR) which can be subtracted from the raw data to allow easy re-referencing to a common average reference scheme.

### Trial information (intervals_trials)

The continuous data can be epoched using event markers (start_time). These markers index either stimulus onset times (articulation = 0) or articulation onset times (articulation = 1). Trials contaminated by inter-ictal epileptic spikes or disrupted with behavioural distractions are marked (good = 0). Trials with successful identification of the presented landmark were marked (correct = 1). Stimuli presented were either coherent images (scrambled = 0) or scrambled images (scrambled = 1) and the exact stimulus presented is noted (stimulus). JPEG versions of the presented stimuli are available for download alongside the datasets.

## Technical Validation

### Behavioural analysis

**Fig. 2:**
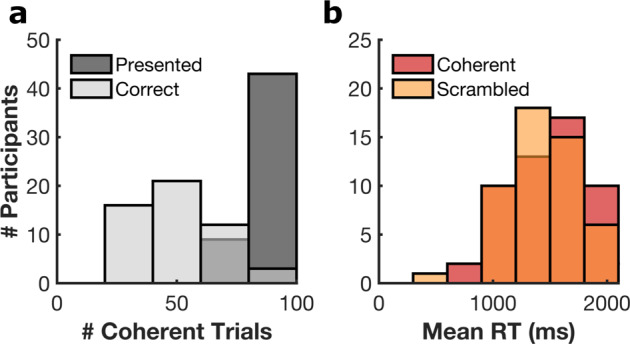
Behavioural Analysis. (**a**) Number of coherent images presented per participant and the distribution of number of successful naming trials. (**b**) Response time (RT) distributions for correctly answered responses for coherent and scrambled trials.

Mean identification accuracy (±s.d.) was 56 ± 17% for scenes and 97 ± 5% for scrambled images. All patients included in this dataset correctly identified at least 20 of the scene stimuli and responded to at least 80% of the scrambled trials, providing enough trials for both successful and unsuccessful identification analysis. Articulation latencies for this cohort were 1,495 ± 330 ms for scenes and 1,491 ± 341 ms for scrambled images (Fig. 2b).

### Spatiotemporal mapping of cortical activations

**Fig. 3:**
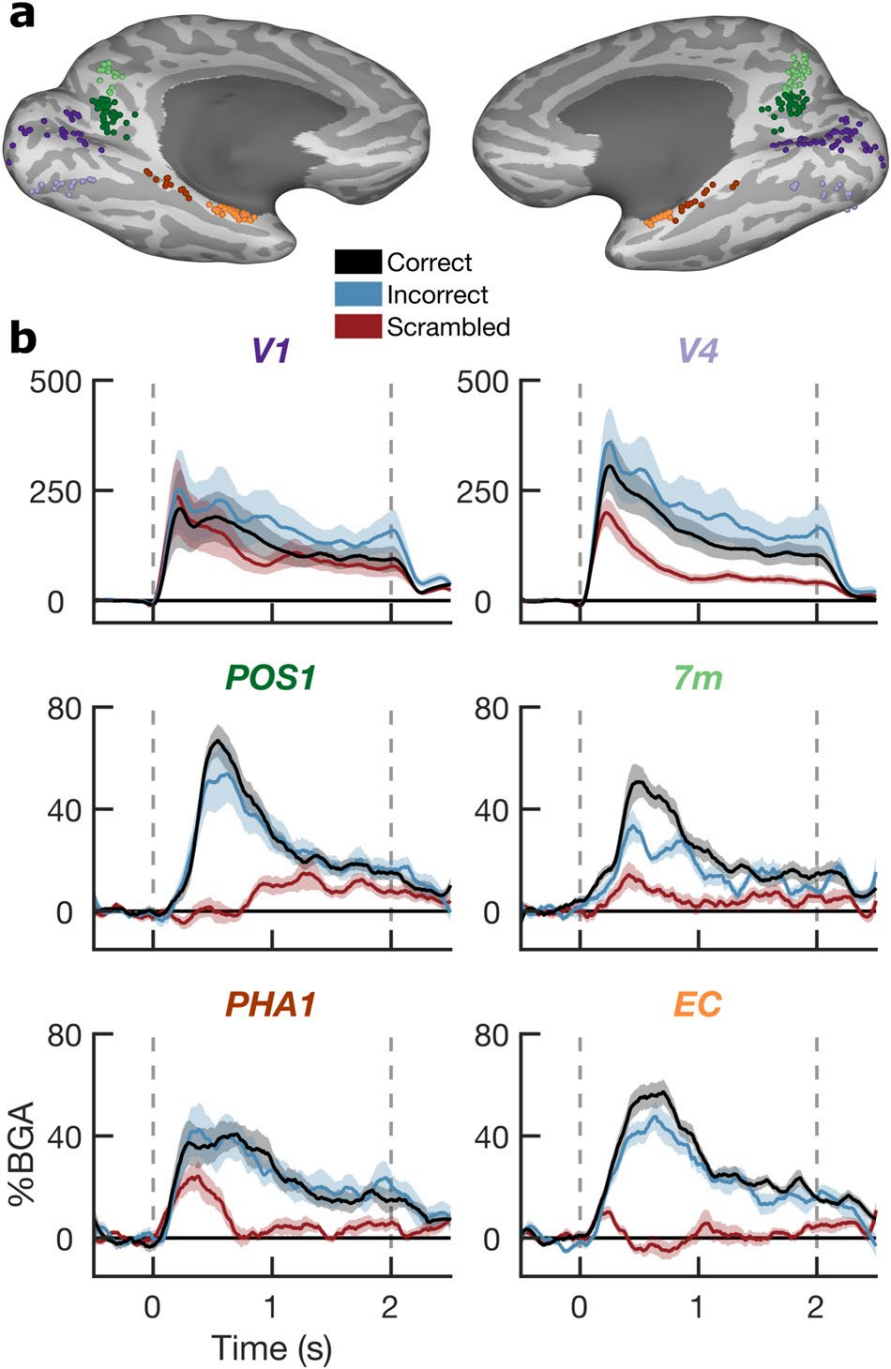
Spatiotemporal Profile of Cortical Activations. Locations (**a**) and BGA activations (**b**; mean ± s.e.) of six ROIs based on the HCP parcellation. V1 (Primary visual cortex; 84 electrodes, 15 patients), V4 (Fourth visual area; 56 electrodes, 12 patients), POS1 (Parieto-occipital sulcus area 1; 69 electrodes, 27 patients), 7 m (Area 7 m; 56 electrodes, 20 patients), PHA1 (Parahippocampal Area 1; 27 electrodes, 14 patients), and EC (Entorhinal cortex; 60 electrodes, 25 patients). Vertical dashed lines denote stimulus onset and offset times.

We have previously used some patients from this dataset to create a 4D movie of whole brain cortical activation, contrasting correctly answered coherent scenes vs. scrambled images^15^ as a broad map of cortical activity during the task. Here, we present plots of BGA for correctly answered, incorrectly answered and scrambled trials across six exemplar ROIs, using HCP parcellations in early visual cortex, medial parietal cortex, and medial temporal lobe (Fig. 3). These ROIs display diverse response properties in their responses to coherent vs. scrambled images and correctly vs. incorrectly identified stimuli.

## Usage Notes

Electrodes are named according to clinical convention within our site, based on the broad location covered (SDEs) or the approximate distal electrode target (sEEGs). This nomenclature scheme is for clinical purposes and may not always reflect actual electrode location. sEEG electrodes are numbered sequentially along the probe with the most distal (deepest in the brain) contact being electrode 1.

Articulation times have only been included for correctly answered trials. Pairing stimulus onset and articulation onset markers should be performed by finding matching stimuli, as each stimulus was shown only once per patient. Baselining for articulation aligned data should be performed using the pre-stimulus baseline period rather than the pre-articulation period.

Electrical time series traces are provided as output from the NeuroPort recording system with no additional filtering applied beyond the acquisition filter (0.3–500 Hz). To convert the traces into μV the values should be divided by 4, after conversion from integers to floating-point values.

Users who wish to publish results of analysis performed on this data must include the following in their acknowledgements: “Data used to perform this analysis were collected with support from the National Institutes of Health under award numbers DC014589 and NS098981 and were accessed from the Data Archive for the BRAIN Initiative with support from the National Institutes of Health under Award Number R24MH114796.”

Access to the data is contingent on creating an account with DABI and agreeing to the terms of the data usage agreement (Supplementary Information).

## Supplementary information

Supplementary Information

## Data Availability

Example scripts are provided with the dataset^25^. They contain code for reading and plotting the neural and behavioural data. Code examples are provided in Matlab and Python Jupyter Notebooks.
