# Supplementary material for: Dataset of human intracranial recordings during famous landmark identification
Source: Sci Data. 2022 Jan 31;9:28. doi: 10.1038/s41597-022-01125-8 (PMC8803828; doi:10.1038/s41597-022-01125-8)
Supplement: Supplementary file 1 — Supplementary Information [file 41597_2022_1125_MOESM1_ESM.pdf]

Houston Intracranial Landmark Identification Dataset Data Usage Agreement
Version 1.0.0

The Tandon Lab at the University of Texas Health Science Center at Houston (UTHealth) wishes to make data available for research and educational purposes to qualified requestors, but only if the data are used and protected in accordance with the terms and conditions stated in this data usage agreement.

It is hereby agreed between the data requestor, hereinafter referred to as the "LICENSEE", and UTHealth, that:

1. The LICENSEE will not attempt to identify any individual referenced in the provided data.
2. The LICENSEE will exercise all reasonable and prudent care to avoid disclosure of the identity of any individual referenced in the data in any publication or other communication.
3. If the LICENSEE finds information within the data that they believe might permit identification of any individual, the LICENSEE will report the location of this information promptly by email to eliana.klier@uth.tmc.edu, citing the location of the specific information in question.
4. The LICENSEE will exercise all reasonable and prudent care to maintain the physical and electronic security of the data.
5. The LICENSEE will not share access to the data with anyone else or redistribute the data to anyone else without prior approval.
6. The LICENSEE will use the data for the sole purpose of lawful use in scientific research and no other.
7. The LICENSEE agrees the data will not be used for any commercial purposes or used for the development of commercial intellectual property.
8. The LICENSEE agrees to contribute code associated with publications arising from this data to a repository that is open to the research community.
9. The LICENSEE agrees to include the following text in the acknowledgements of any publications arising from analysis of the data:

   "Data used to perform this analysis were collected with support from the National Institutes of Health under award numbers DC014589 and NS098981 and were accessed from the Data Archive for the BRAIN Initiative with support from the National Institutes of Health under Award Number R24MH114796."

10. This agreement may be terminated by either party at any time, but the LICENSEE's obligations with respect to the data shall continue after termination.

THE DATA ARE PROVIDED "AS IS", WITHOUT WARRANTY OF ANY KIND, EXPRESS OR IMPLIED, INCLUDING BUT NOT LIMITED TO THE WARRANTIES OF MERCHANTABILITY, FITNESS FOR A PARTICULAR PURPOSE AND NONINFRINGEMENT. IN NO EVENT SHALL THE AUTHORS OR COPYRIGHT HOLDERS BE LIABLE FOR ANY CLAIM, DAMAGES OR OTHER LIABILITY, WHETHER IN AN ACTION OF CONTRACT, TORT OR OTHERWISE, ARISING FROM, OUT OF OR IN CONNECTION WITH THE DATA OR THE USE OR OTHER DEALINGS IN THE DATA.

NAME: ____

INSTITUTION: ____

SIGNATURE: ____

DATE: _________________________

Please return the signed form to Nitin Tandon (nitin.tandon@uth.tmc.edu)
